# Phenomenological support for escape theory: a qualitative study using explicitation interviews with emotional eaters

**DOI:** 10.1186/s40337-022-00690-y

**Published:** 2022-11-21

**Authors:** Huma Shireen, Samantha Castelli, Maurice Legault, Yair Dor-Ziderman, Julia Milad, Bärbel Knäuper

**Affiliations:** 1 Department of Psychology, McGill University, 2001 McGill College Avenue, Montreal, QC H3A 1G1 Canada; 2 Department of Teaching and Learning Studies, University of Laval, Quebec City, Canada; 3 Edmond J. Safra Brain Research Center, University of Haifa, Haifa, Israel

**Keywords:** Emotions, Eating, Phenomenology, Qualitative, Emotional eating

## Abstract

The current study explored the phenomenology of emotional eating, that is, the descriptive knowledge of what one perceives, senses, and knows in one's immediate awareness and experience during emotional eating. Eight individuals with emotional eating were interviewed twice using explicitation interviewing. Data were analyzed using thematic analysis, which resulted in nine themes describing the diachronic (or temporal) unfolding of emotional eating and several sub-themes that described various synchronic (or experiential) dimensions of this unfolding. The core findings of this study support the escape theory of emotional eating and recommend future directions to investigate the self-related shifts proposed by this theory. Namely, the findings show that individuals tend to use food to regulate their emotions by reducing the unpleasant experience of negative emotions and the associated unpleasant narrative processing or ruminations about stressors that caused the negative emotions. This then leads to an urge to eat associated with a desire for the sensory experience of eating. Eating then enables individuals to reduce thoughts about their stressors and bring themselves into the present moment through embodiment. Future quantitative research could investigate this mechanism of shifting from narrative to embodied processing to regulate emotions in emotional eating to develop treatment programs, such as mindfulness-based programs, that could encourage such a shift and emotion regulation without the use of food.

## Plain English summary

Emotional eating, or overeating when feeling negative emotions, is problematic because it is linked with being overweight or having obesity, diabetes, and heart disease. So far, several theories have been proposed to explain the psychological experiences of a person that leads them to emotionally eat. In this study, we explored the subjective experience, or phenomenology, of emotional eating. We interviewed eight individuals with emotional eating using a method called explicitation interviewing, which was developed to allow an interviewer to help an individual describe their subjective experience or phenomenology of a particular event or experience. Upon analyzing the data, we found support for the escape theory of emotional eating, which states that individuals eat to regulate their emotions by escaping the unpleasant feelings and thoughts that they experience while feeling negative emotions. We found that emotional eaters escape unpleasant thoughts and feelings about their past, future, or identity by shifting their attention away from these thoughts and emotions and towards the bodily sensations that they feel in the present moment while eating. Our findings encourage future studies that further investigate these shifts in attention to develop treatment methods that can help individuals regulate their emotions without using food.

## Introduction

Emotional eating is defined as “the tendency to overeat in response to negative emotions, such as anxiety or irritability” [58], p. 106. Such overeating generally involves consuming foods that are high in sugar and fat and can lead to an increased risk of weight gain, negative body image, and compensatory behaviors, such as overexercising to lose weight [18]. Given these negative health outcomes of emotional eating, several mechanisms of emotional eating have been proposed and investigated using a variety of methodologies to develop targeted interventions for this behavior [40, 52, 58, 62]. Thus far, only a handful of studies have investigated the first-person subjective experience, or phenomenology, of individuals as they emotionally eat. Such phenomenological explorations have been successfully used in other lines of research, for example, in investigating the precursors of epileptic seizures to allow individuals to better anticipate them [46] and in understanding the mental correlates of meditation to improve the way in which it is taught and practiced [47, 48]. The aim of the current study is to undertake such a phenomenological exploration of emotional eating to add to our understanding of how emotions influence eating behavior and the role played by food in emotional eating.

Thus far, several theories of the mechanisms underlying emotional eating have been proposed. For example, the psychosomatic theory posits that poor interoceptive awareness results in an inability to recognize hunger and satiety and distinguish these signals from other bodily sensations, including emotional arousal, leading to eating in response to emotional arousal [8]. Similarly, the restraint theory posits that negative affect triggers overeating specifically among people who are restrained eaters because of a temporary disinhibition of self-control and cognitive effort required to resist the desire to eat [28]. Another popular theory of emotional eating is the escape theory by Heatherton and Baumeister [27] who propose that individuals overeat in response to negative emotions to attempt to escape or shift away from ego-threatening stimuli that cause aversive self-awareness and instead focus on salient external stimuli, such as food. Research support for this theory comes from studies that have shown increased food intake in individuals with emotional eating following stressors that are regarded as ego-threatening, such as public speaking [44], or tasks using emotionally laden words that were perceived as ego-threatening (e.g. inadequate, ridiculed, abandoned, failure, etc., [66]).

Thus far, research testing these theories has used a variety of methods such as self-report questionnaires, laboratory studies, and testing in naturalistic settings [40, 52, 58, 62]. Recently, Bongers and Jansen [5] conducted a literature review on the different methods used to investigate emotional eating and concluded that a number of these methods have limitations that render them ineffective at explicating the processes underlying emotional eating. For example, these authors questioned the validity of self-report questionnaires that assess emotional eating by citing research wherein high scores on these questionnaires may reflect various other constructs such as a lack of control, general eating concerns, a tendency to attribute overeating to negative affect, or reactivity to food cues [1, 31, 57]. They also questioned studies that assess food intake after the induction of different moods, for example by having participants watch emotionally laden film clips, recalling a personally relevant emotional event, or completing a social stress task. Specifically, the authors highlighted that some of the results of these mood induction studies showed no difference in the amount of food eaten between individuals with and without emotional eating and at times even showed increased eating in individuals with emotional eating in neutral mood inductions [15, 44, 61]. Based on these mixed findings, Bongers and Jansen [5] argue that these mood induction lab paradigms may be unsuccessful in measuring emotional eating because they do not accurately reflect the experience of emotions or eating in real life. Furthermore, Bongers and Jansen [5] state that naturalistic studies of emotional eating, which aim to assess real-life emotions and eating behaviors, have also shown similar mixed results. Other authors have speculated that one core reason for the mixed findings of naturalistic investigations of emotional eating may be due to the complex relationship between emotions and eating due to confounding variables such as external eating, eating alone versus in company, or food availability, that influence emotional eating but are not addressed by most naturalistic methods [7, 49].

In addition to these methodological limitations, several researchers working outside the realm of emotional eating have emphasized that the scientific study of psychological processes is incomplete without the careful integration of data targeting participants’ subjective experiences with the more widely available self-report questionnaires and behavioral data [10, 21]. In emotional eating research, self-report questionnaires measure individuals’ thoughts, beliefs, or judgements related to emotional eating but do not capture the descriptive and embodied aspects of their subjective experiences while emotional eating. Similarly, behavioral measures capture observable variables related to emotional eating without providing much information on underlying psychological processes. Other research has relied on qualitative methodology to provide first-person subjective data on emotional eating. For example, Tuncer and Duman [55] used a descriptive qualitative design to find that negative emotions acted as triggers for overeating that led to the experience of positive emotions but also a loss of control. Similarly, Wehling and Lusher [65] conducted semi-structured interviews with individuals either at a healthy weight range or above to find that participants’ relationship with food was emotionally charged and that links between dieting and negative thinking fostered unhealthy eating patterns. Even though these studies add to our understanding of processes underlying emotional eating, they primarily target individuals’ thoughts *about* their emotional eating without directly accessing individuals’ structures of consciousness as they emotionally eat. Directly targeting and studying these structures of consciousness during individuals’ emotional eating experience may allow us to add to our knowledge about emotional eating that is not accessible through other methods. One way to access this information is through the study of phenomenology, defined as “knowledge as it appears to consciousness, the [description of] what one perceives, senses, and knows in one's immediate awareness and experience” [42], p. 26. Thus, the focus of this study is to use a qualitative methodology to explore the phenomenology of emotional eating. Specifically, the current study addresses the following questions about the phenomenology of emotional eating: Which aspects of an emotional experience do individuals avoid using food? What are the needs of individuals, unrelated to food, during these emotional experiences? What are some obstacles to meeting these needs without eating? And how does the experience of eating help in coping with emotions?

## Method

The study was approved by the McGill University Research Ethics Board (REB# 21-09-046) and therefore is in accordance with the ethical standards laid down in the 1964 Declaration of Helsinki and its later amendments. All participants provided informed consent before participation in any study procedures.

### Participants

Participants in the current study were recruited based on the following inclusion criteria: adults (at least 18 years old); mean score of at least 3.25 out of 5 on the emotional eating subscale of the Dutch Eating Behavior Questionnaire (DEBQ) [59], often used to identify emotional eaters in research contexts [60], and able to read and understand English. The DEBQ consists of 13 items such as “Do you have a desire to eat when you are depressed or discouraged?” and “Do you have a desire to eat when you are approaching something unpleasant to happen?” The exclusion criterion was the presence of self-reported psychiatric illness, including any eating disorders. Participants were recruited through advertisements in online community forums on social media platforms and through McGill University student forums and staff and alumni newsletters. Interested individuals were asked if they tend to overeat in response to negative emotions to confirm their tendency to emotionally eat. This question was based on Van Strien et al.’s [59] definition of emotional eating, i.e., overeating in response to negative emotions such as anxiety, stress, sadness, boredom, anger, etc. Individuals who endorsed this behavior were then invited to the screening portion of the study, where they completed the DEBQ to confirm their emotional eating. Purposeful sampling, a non-probability-based sampling method aimed at recruiting members from specific populations, was used to include participants from diverse ethnic populations. This was done by selecting participants from diverse ethnic backgrounds from the pool of the individuals who showed interest in participating in our study. All interviews were conducted in English.

Eight participants (four men and four women) were included in this study. Participants in this study ranged from 19 to 52 years of age and their average score on the emotional eating subscale of the DEBQ was 3.75. Of the eight participants, four reported a BMI in the overweight range and two reported a BMI in the obese range. A summary of all participants’ demographics information is provided in Table 1. Details of the participants and summaries of their experiences are included in a supplemental document (see “participant summaries”). All participants are identified by pseudonyms for confidentiality.

**Table 1:** Participant demographics

Name	Age	Gender	Location	Employment status	DEBQ- emotional eating subscale score	BMI	Ethnicity	Highest education
Stephanie	52	Female	Montreal	Freelance	3.76	28.19	North American, European	Undergraduate studies
Raj	29	Male	Montreal	Graduate student	3.92	24.12	South Asian	Graduate studies
Adam	49	Male	Montreal	Full-time	3.54	27.89	North American, Eastern European	Undergraduate studies
Ross	30	Male	Toronto	Full-time	3.3	31.66	European	Undergraduate studies
Steve	19	Male	Montreal	Undergraduate student	4.15	25.10	European	High school or equivalent
Ruth	35	Female	Quebec City	Full-time	3.46	33.83	North American Indigenous, European	Graduate studies
Fatima	39	Female	Montreal	Full-time	4.46	29.23	North African	Vocational or technical training
Sofia	20	Female	Montreal	Part-time student	3.38	22.63	African-Caribbean, Latin American	Undergraduate studies

### Interview method

Data in the current study were collected using explicitation interviewing (EI) by H. S., the first author of this study, and M. L., a co-author of this study and a certified trainer of EI [37, 38]. EI is a method developed to enable individuals to access and verbalize the phenomenology of an event [63] by not only cognitively recounting a memory but by reliving it in a sensorily embodied manner, through the *embodied speech position*. The embodied speech position is thought to reduce bias in memory recall and allow interviewees access to aspects of their experiences that they were previously unaware of [64]. The interviewers enabled the embodied speech position by guiding participants to relive their experiences of emotional eating by describing the temporal sequence of their actions during the event. These temporal aspects of experience are called the diachronic dimensions of participants’ experience [47, 48]. Additionally, participants were guided to re-situate themselves in the physical environments of the event by sharing details of their environmental surroundings during the event. At each moment of interest in the diachronic dimensions of the experience of emotional eating, the interviewers focused the participants on granular details of their lived embodied experience rather than on their interpretations or judgements of the experience. These granular details included sensorial information, emotional experiences, and attentional tendencies, related to the synchronic dimension of experiences [47, 48]. In exploring these diachronic and synchronic dimensions of experience, the interviewers phrased the questions in the present tense to further enable participants to relive their experiences in the present moment [14]. The embodied speech position is thought to be recognizable when interviewees respond in the present tense in addition to showing other markers such as movement of gaze away from interviewer and slowing down of speech [63]. Furthermore, participants may also use gestures to convey embodied reliving of pre-reflective information that is currently verbally inaccessible to them. In keeping with the EI methodology, the interviewers paid close attention to the use of these gestures and brought the participants' attention to them, thus enabling the participants to describe experiences related to these gestures [45]. Throughout the interview process, the interviewers followed the EI method of preventing the induction of content in the participants' subjective experiences by asking short and open-ended questions (e.g., “what do you do next?”). In addition, the interviewers used the participants' own words for subsequent questioning (called Ericksonian reformulations [23]), a method that is in keeping with *bracketing* in phenomenological research, or the “deliberate putting aside one's own belief about the phenomenon under investigation or what one already knows about the subject prior to and throughout the phenomenological investigation” [11], p. 2. Finally, the interviewers attempted to maintain awareness of their own internal psychological processes during the interview to differentiate them from those of the participant. For example, the interviewers remained mindful of their own emotional reactions through their bodily sensations and their thoughts and tendencies to want to explore aspects of participants’ experiences, including clinical aspects, that were not directly related to the research questions. By becoming aware of these processes and not following them, the interviewers were able to remain focused on the experiences of the participants and the goals of the study during the interviews. All participants described one experience per interview that occurred within two months of the interview, except for one participant (Adam) who recalled an experience that occurred four months ago.

Some examples of questions that were asked during the interviews are:

1. To identify the emotion that leads to eating: What do you feel in this moment? Where in your body do you feel it? How does it feel? What do you want to do in response to it? What is it about the emotion that makes you want to do this? What is this emotion telling you about yourself?
2. To identify the psychological need: What does it feel like the emotion needs? What does it feel like you need in this moment? How do you want to give it/yourself that? What would that do for you? How would you feel? What does this new feeling say about yourself?
3. To identify the role of food: What do you feel when eating? Where in your body do you feel it? How does it feel? What do you want to do in response to feeling this emotion? What does this feeling tell you about yourself?

### Procedure

All interviews were conducted via Zoom due to restrictions related to the COVID-19 pandemic. Each interview lasted for approximately one hour. Each of the eight participants was interviewed twice (one week apart), resulting in approximately 16 h of interview data, which is comparable to the sample size of other studies that have used the method of EI [3, 36]. Two interviews were conducted for the following reasons: a) to allow for the adaptation and personalization of questions asked in the second interview to more deeply explore the specific participant’s phenomenology of emotional eating, b) to allow participants to learn how to successfully engage in an explicitation interview to increase the ease with which they can access their phenomenology of emotional eating in the second interview, c) to validate the within-participants findings from the first interview, and d) to examine whether and how the self-reflection encouraged in the first interview influenced the participants’ emotional eating. At the end of the second interview, the audio and video files were transferred to password protected computers to ensure confidentiality. Transcription of all interview data was done through the software ‘Otter’ and was checked independently by the first and second authors for accuracy.

### Data analysis

Data analysis focused on portions of the interviews that included experiential aspects of individuals’ experiences during specific moments of emotional eating while excluding contextual information, descriptions of general experiences of emotional eating rather than details of a specific event, or thoughts, beliefs, or judgements about emotional eating. Data were analyzed using thematic analysis using QSR International’s NVivo-12 software (2018). First, H. S., who has previous experience in qualitative research [50], trained S. C. in coding lines of the interview to calibrate this process. Both H. S. and S. C. then independently coded the interviews. All coding was done using gerunds (i.e. words ending in ‘ing’), based on a method known as process coding, to identify actions and processes within the interview data [67]. Additionally, an inductive approach was taken to link the data with appropriate codes rather than to pre-existing theories and to continuously compare codes to each other to minimize repetition. Once the independent coding was completed, all codes were compared by the two authors. A small number of disagreements arose (on approximately less than 5% of the codes) that were resolved by discussion to reach a mutually satisfactory understanding of the phenomenology being described by the participant and the code needed to label each experience. Following this stage of independent coding and discussion, H. S., S. C., and J. M. collectively grouped the codes into themes and compared the themes to one another to establish relationships between them. These themes were created to capture a meaningful construct in relation to the overall research question, representing a level of patterned response within the data set. Finally, the themes were created and organized in a diachronic manner representing the temporal dimension of emotional eating experiences as they unfolded.

### Validity of the study findings

In addition to conducting two interviews to validate within-participant data, further measures were taken during the data analysis stage to increase the validity of data. Firstly, as mentioned, all authors involved in the data analysis completed the coding independently and discussed their findings at the end to reach a consensus and minimize errors. Secondly, J. M. who was involved in the data analysis during the stage of theme development, was naïve to the study data and was able to provide additional input on the accuracy of developed themes and their relevance to the research questions. Finally, all authors involved in the data analysis kept journals throughout the process to mitigate their biases, which is another method that enables bracketing in phenomenological research [11]. For example, some of the bracketed beliefs were that all individuals are aware of their negative emotions, that negative emotions are always experienced as aversive, and that all emotions are experienced as bodily sensations only. When relevant, the authors discussed their biases and its impact on the data analysis. Only those codes and themes that were judged by all authors to be free of bias and relevant to the research questions were retained.

## Findings

**Fig. 1:**
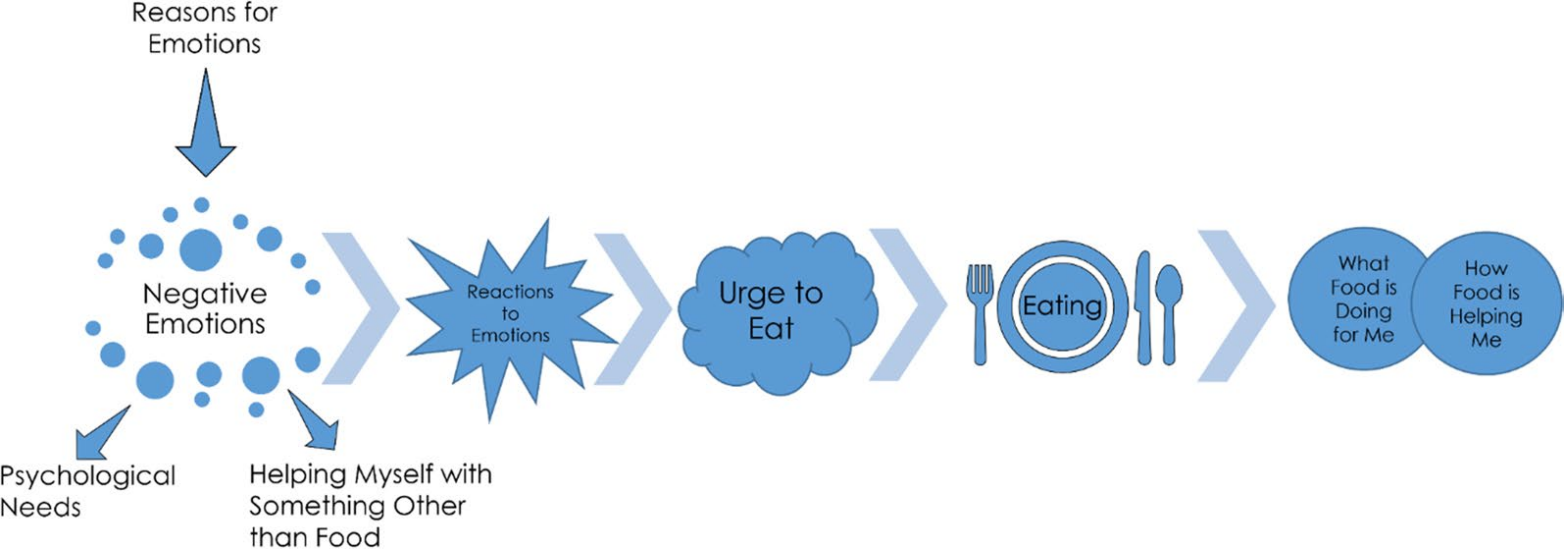
Study themes: diachronic unfolding of the phenomenology of emotional eating

The thematic analysis of the data resulted in the following nine themes (summarized in Fig. 1) delineating the diachronic dimension of the participants’ emotional eating phenomenology: “negative emotions”, “reasons for emotions”, “psychological needs”, “reactions to emotions”, “helping myself with something other than food”, “urge to eat”, “eating”, “what food or eating is doing for me”, and “how food or eating is helping me”. The core findings of this study are contained within the sub-themes, which describe the synchronic details of participants’ experiences during emotional eating. The study themes, sub-themes, sample quotes, and the number of participants who shared each sub-theme are summarized in Table 2. Additionally, Table 2 identifies the sub-themes that emerged in relation to the questioning used in this study (identified with an “*”) and ones that emerged from participants independently sharing details of their experiences. The core findings are outlined below.

**Table 2:** Study themes, sub-themes, number of participants, and sample quotes

Themes (diachronic events)	Sub-themes (synchronic details)	Participants	*n*	Sample quotes
Negative emotions	Types of emotions*	Stephanie, Raj, Adam, Ross, Steve, Ruth, Fatima, Sophia	8	Adam: “Um, I was very sad. It's sadness definitely. I mean almost a bit of loss because it's ending that relationship, sort of, with this band. I've been doing it since 1994”. Steve: “My brain really starts to, like, just become more anxious”.
	Feeling it in the body*	Stephanie, Raj, Adam, Ross, Steve, Ruth, Fatima, Sophia	8	Raj: “From what I remember the heart went up racing. You know feeling of anxiety. The heart was racing up like thinking of all the work I would have to do on it again, senseless work”. Sophia: “From my body. It’s just like my head. It would be like a bit of everywhere”.
	Lingering emotions	Raj, Adam, Ross, Sophia	4	Adam: “I'll beat myself up with for quite a while, you know, for the rest of the day at least and then, you know, it stays with me a bit”. Ross: “… don't get me wrong, there's still a lot of baggage to deal with over the next couple of days…”
Reasons for emotions	External factors	Stephanie, Raj, Adam, Ross, Steve, Ruth, Fatima, Sophia	8	Steve: “… a lot of it comes down to COVID and isolation, a bit like here in my building, in my residence, there was a COVID outbreak, and it was just like everybody had to stay inside”. Ross: “… some hurtful things towards me where it kind of implied that [my father] thinks I didn’t help out enough. And that was really difficult for me to not only hear but also to process. So, I rather lashed out at him today [saying], ‘that's completely unfair, I spent my entire day here with you doing X, Y, and Z. And now you're being rather unfair. So, I'm leaving’”
	Psychological processes	Raj, Adam, Ross, Steve, Ruth, Sophia	6	Raj: “I have this stress in my head, “what's going to happen in the future?” Whether I'm going to find a tenure track position”. Steve: “… like just trying to find some … like a lack of urgency in my life right now. I think that lack of urgency is definitely accentuated by COVID-19. There's just nothing really to work for right now”
	Ways of relating to myself*	Stephanie, Raj, Adam, Ross, Steve, Ruth, Fatima, Sophia	8	Adam: “I've sort of wasted my time again. I went it through this once. I went through it again. And I'm a little like I'm older now and I shouldn't waste my time with these sorts of things. ‘Why do I do this?’” Sophia: “And once I was over there. I found myself being angry and with a lot of impotence instead, being anxious and sad”.
Psychological needs	Behavioral, interpersonal, or self-related needs*	Stephanie, Raj, Adam, Ross, Steve, Ruth, Fatima, Sophia	8	Adam: “I needed a break from it. So, it was trying to … not just physically typing and you know being online but the thinking about it and everything else”. Ross: “… I also need to be able to be okay with redefining those moments. I think what Ross prior to that last year in university would have defined those spark moment or the passion as might not be the same definition as the Ross today”
	What meeting needs would do for me*	Stephanie, Raj, Adam, Ross, Steve, Ruth, Fatima	7	Steve: “… I could do so much better in terms of like spending half the amount of time doing my work and doing greater things in life”. Ross: “… unlock and liberate me to redefine my passions and those spark moments”
	Feelings, beliefs, and self-related reasons for not helping myself*	Stephanie, Raj, Adam, Ross, Steve, Ruth, Fatima, Sophia	8	Stephanie: “It’s taking the time to do it. It’s almost as if I’m saying like, ‘Oh, well, I’m not really worth it. This is not really worth taking the time to do, like, who cares?’” Raj: “I don’t want to be going out every day and buying Chèvre [cheese] just to feel like that because I have many people around me who are obese and who make excuses [and say] that I am perfectly fine. I don’t need to do that”.
Reactions to emotions	Wanting emotions gone	Stephanie, Adam, Fatima	3	Stephanie: “The desire to escape more. The desire to not address my feelings. The desire to not deal with things”. Adam: “that's why I like sleeping because it just shuts everything off, you know, I don't have to think it's just not. It's just a way of shutting down the brain”
	Having mixed reactions to emotions	Ross, Ruth, Fatima	3	Ruth: “I love my job. I feel so lucky that I got this appointment. But sometimes, it's a lot. And I just wanted to leave and go back home”
	Thinking a lot	Raj, Adam, Ross, Steve, Sophia	5	Ross: “Like when I’m with myself too long, my inner monologue definitely gets louder and makes me just start to critically evaluate every single thing I’m doing”. Steve: “I think my, my inner monologue got very loud, because I wasn't talking to anybody”
Helping myself with something other than food	Behavioral ways	Raj, Adam, Ross, Steve, Ruth, Fatima, Sophia	7	Ross: “So you know you’re driving away from the driveway, waving at her, telling her ‘Hey, I love you,’ but also just saying ‘Hey, you know, I kind of want to get into my own space right now.’” Ruth: “And it's even less prevalent ever since I quit social media. So, there's even less of a measure of comparison going on”
	Interpersonal ways	Raj, Adam, Ross	3	Raj: “The first thing I thought, that came in my head after that was, ‘I need to call my brother because he will counsel me.’” Ross: “I see a counselor that I speak to me about a couple month basis here and there. And one of the things that we've decided that I think is really important for me to work on… is being aware of what's going on in that moment, not being reactive”
	Mental ways	Raj, Adam, Ross, Steve, Ruth, Sophia	6	Raj: “And I try to make myself realize each and every day that all this … this professor, this career thing that I’m doing is … it’s not going to last forever”. Sophia: “… it’s already here. It’s happening”
	Positively relating to myself	Raj, Ross, Steve, Sophia	4	Ross: “… this time around, you’re going to fight all the natural urges. You do have to really try and set yourself up for success” Steve: “And then I went into full gear and said, okay, time to get this done, because it's due at midnight and I did get basically everything done”
Urge to eat	Reasons for wanting to eat*	Stephanie, Raj, Adam, Ross, Steve, Ruth, Fatima, Sophia	8	Stephanie: that “… there comes that, you know, desire to fill that emptiness”. Ross: “[I thought], ‘You just worked hard the entire day, you weren’t appreciated, and then on top of that now you’re upset.’ For whatever reason, the initial response within me was ‘What is going to give you an enjoying relaxing night to go home to, get away from all that?’
	Looking forward to the sensory experience	Stephanie, Raj, Adam, Ross, Ruth	5	Raj: “It tastes like chocolates, like the creamy flavor chocolate ice cream but sometimes you get the chocolate chip inside. And, yeah, it's something else”. Ross: “But I definitely was clicking in associating with that first moment of enjoyment… of putting the product in my mouth and enjoying it, tasting it. So that is what kind of drew me to it. That's what kind of got me going”
	Automatically going towards food	Raj, Steve, Ruth, Sophia	4	Raj: “I remember that when I went to Walmart, I was just saying to myself, ‘You don’t want to buy it. [My girlfriend] is also not interested in buying it. Why are you looking at it?’ Still, I went to Walmart”. Sophia: “I truly cannot pinpoint something specific that made me just go… And I bring it right next to my table back again and it was very quick. And I was over there eating”.
	Wanting convenient and specific foods	Raj, Adam, Ross, Steve, Ruth, Fatima, Sophia	7	Adam: “And it would have just been like, ‘What am I going to do tonight? I’m just going to order.’ There’s something about the convenience”. Ross: “I can recollect now that I reflect upon it, and I knew in that moment, those Reese’s Pieces were going to be mine at one point”
Eating	Overeating	Stephanie, Adam, Ross, Steve, Ruth, Sophia	6	Stephanie: “… and then I went back in. Even though I wasn’t hungry anymore, I had another scoop. Another small scope of the lentils”. Steve: “I'll have some nuts now I'll have somebody you know one thing leads to another”.
	Positive feelings while eating *	Stephanie, Raj, Adam, Ross, Steve, Ruth, Fatima, Sophia	8	Adam: “I guess to me, and thinking about it, you know, a mom’s hug or something like that… there’s something very nurturing about it and comforting”. Ross: “I think this unwinding. It feels like per bite you feel more and more relaxed, per bite you feel more and more unwound, per bite you feel more and more away from the stress that you’re under that day”.
	Enjoying the sensory experience	Stephanie, Raj, Ross, Steve, Sophia	5	Ruth: “The only reason I’m here is because I don’t want to be doing my work, and this is like tasting good and making me feel good”. Raj: “It feels really good. I just want to close my eyes and enjoy that taste”.
What food or eating is doing for me	Coping mechanism	Stephanie, Ruth, Sophia	3	Ruth: “So it's not like an experience is just a coping mechanism, and I can feel it at that that moment”
	Rewarding myself	Ross, Ruth, Fatima	3	Fatima: “Long day at work, come back home, and then I sit down like ‘Ah. Okay, now, after I did all of that I deserve that reward.’”
How food or eating is helping me	Helping me with work	Ruth, Steve, Sophia	3	Steve: “Because something about getting some food and making a little bit of food, even if it's just a small little bit like actually can let my brain take a break”
	Giving me something to do	Raj, Fatima, Sophia	3	Raj: “But, yeah, that's I think that's one of the reasons why I'm sipping on coffee again and again I don't know, just trying to keep myself busy”
	Positively influencing my feelings	Raj, Adam, Ross, Steve, Fatima, Ruth	6	Raj: “I would like to have two or three days, let's say, in a month, where I can just eat and take out the stress”. Ross: “And because of that I said to myself, ‘you know what, it's late, I don't really care right now, I'm gonna take control and I'm going to say, for me, I'm going to go have a snack.’”
	Not thinking about my stressors	Raj, Adam, Ross, Steve, Ruth	5	Raj: “… giving me something else to think about, the experience of eating, the taste, the smell, everything. So, it's just less focus on that one thing”. Ruth: “It just helps me to think about something else”
	Being in the moment	Raj, Ross, Ruth, Sophia	4	Ross: “And man, you're going to have another whopping day at work tomorrow. But for right now, you here with the burger… just enjoy this”. Sophia: “I can sit down over here but if I had my coke next to me, that would be amazing… just in the moment, having my music back and trying to write something. It will just make me feel great”

*Sub-theme was closely related to questions used to guide participants

### Negative emotions

All participants were guided to describe their experience of negative emotions that lead to emotional eating. The EI methodology enabled several participants to access layers of their emotional experiences that they were previously unaware of. Stephanie exemplified this in saying the following:

It just came to me now. Maybe I wasn't fully aware of it last night, but just saying it now that, you know, ‘how did I feel at that moment when I was going to get more food?’ Now I feel like I'm starting to feel teary eyed. Yeah, sadness.

Participants described the different types of aspects of their experience of negative emotions in the sub-themes below.

#### Lingering emotions

Four participants described their negative emotions as lingering for a while. For example, Raj stated: “There is no end of that moment. That stress stayed with me I think … for one and a half days”. Sofia stated that she didn’t think her emotion “went away at any moment” and that “… it's definitely an emotion that happened throughout the course of the day until it was actually the time of the exam. I don't think it went away at any moment.

### Reasons for emotions

Participants described different aspects of their lives that caused the negative emotions. Some participants described some reasons for experiencing negative emotions that they were previously unaware of. For example, Ross shared the following:

I think for me, now that I sit with it a bit more and I'm being asked these questions, restlessness [comes from] this pushback or want[ing] not to default into old unhealthy tendencies of going back out to that living room.

These different reasons for experiencing negative emotions that led to emotional eating are discussed in the sub-themes below.

#### Psychological processes

Six participants in this study elaborated on internal psychological processes that caused their negative emotions. For example, Ross described his experience of feeling uncertain about his career choices in stating that “… it's easy to eat and stay within that moment of fulfillment, that feeling of control. I maybe didn't get the ability to dictate what I was going to do coming out of high school or university”. Similarly, Adam described the stress he felt due to a work-related change by saying that “It was the stress of having to start working from home, you know, figuring that out. It was a lot of new things as well so that was stressful for me”.

#### Ways of relating to myself

All participants in this study were guided to describe their experience of ‘self’ in relation to their negative emotions. In response, four participants described feeling like they were “not good enough” and seven participants described wanting more from themselves. For example, in describing a sense of frustration with himself, Ross shared the following:

I sort of waste time. Now that I’m older, I went through it once. I went through it again. And I'm a little like ‘I'm older now and I shouldn't waste my time with these sorts of things,’ you know? Why do I do this?

Similarly, in describing that she wanted more from herself, Ruth stated “So there's a feeling of me wanting to perform as well as to finish it. I just don't want to complete whatever task I'm doing. I want to be good at whatever I've done too. So, there's that”.

### Psychological needs

Following a description of their negative emotional experience, all participants in this study were asked about their psychological needs in these moments. Some participants described needs that they had not been aware of in the past. For example, Ruth stated that “I think I need to do something more active. So, I think that would be maybe the realization here”. The psychological needs of participants in the moments of experiencing negative emotions are described in the sub-themes below.

#### Behavioral, interpersonal, or self-related needs

All participants were guided to describe various things they needed to do to help themselves in moments of experiencing negative emotions. For example, Stephanie described needing to leave her physical location and write about her emotions in saying that “I think that would be very beneficial to actually physically remove myself and go downstairs, here in my office and write”. Four participants described the need for social connection to help themselves in response to their negative emotions. For example, Steve stated that “If I were to do work with other people in person. If I were to talk about my work with other people, then that would definitely help”. Three participants described needing to relate to themselves in a certain way. For example, Sofia described needing self-assurance in stating that she needed “… confidence in my work. I think just trust in it. I think that's, that's what I needed at the moment”.

#### What meeting needs would do for me

Participants were then guided to describe what meeting their needs would do for them. Participants descriptions within this theme varied. For example, Stephanie stated that journaling would help her feel that she was “addressing the issue” and that she would not “feel this this pit in [her] stomach anymore”. Adam stated that talking to someone would help him organize his thoughts by stating that “… just because it’s talking about it, helps you in organizing and just realizing it”. Finally, Fatima shared that she would feel “free and alive” if she was able to move to a city that she liked to be in.

#### Feelings, beliefs, and self-related reasons for not helping myself

All participants were also guided to describe what kept them from meeting their needs following their experience of negative emotions. In response, they described different feelings, beliefs, and self-related reasons for not responding to their needs. For example, Ruth stated that “… if I don’t perform, I feel guilt… So maybe my eating behavior, it might be related to guilt”. Similarly, Ruth stated that “[Helping myself] is not an option. I’m at work. I am busy with things so it’s just not something that occurs to me”. Finally, Sofia described being hard on herself, which kept her from helping herself. She stated that “I’m tough myself, I’m a critic of my work. And I’m trying to be myself while being the judge at the same time”.

### Reactions to emotions

Participants in this study also described various psychological reactions to their negative emotions. These are outlined in the sub-themes below.

#### Thinking a lot

Five participants described thinking a lot in response to their negative emotions. For example, Raj shared that “I’m waiting for the response for a job or something, some job responses or something like that. But these are the things that going on in my head and it makes me want to nibble on something”. Similarly, Adam stated that “I definitely really feel that way, just revisiting it. I'm just going over it again”.

### Helping myself with something other than food

Participants in this study also shared that they engaged in activities other than eating to try to help themselves during moments of negative emotions. These methods are described below.

#### Behavioral ways

Seven participants in this study described distracting themselves in response to their negative emotions. For example, Stephanie stated that “I would try to watch something maybe on Netflix or something, eat something… do something around the house. Yeah, keeping myself busy”. Participants also described taking a break to cope with emotions. For example, Sofia shared that “I had to step away from it a little bit. And I’m like ‘okay, I’ll leave it in the living room. I’ll go to my other class and then I’ll see you back.’”.

#### Interpersonal ways

Three participants described talking to either a therapist or with close others about their negative emotions to help themselves. For example, Ross shared that speaking with a therapist has helped him better cope with difficult situations. Similarly, Adam stated that “I did call EAP about counseling too… because I felt I needed to… just to talk to people about it”.

#### Mental ways

Six participants described helping themselves by reminding themselves of similar past experiences or thinking of a greater context of their situation. For example, Ross described reminding himself of a moment in the past and reasoning to himself by saying that “Hey, you’ve been here before. You know what’s going on. You know what this is derived from and what this comes from. Take a moment to acknowledge that”. Participants also described trying to accept their situations to feel better. For example, Steve stated that “… just like in this current building. I know that I’m not able to change it. So, I kind of accept it”.

#### Positively relating to myself

Four participants described utilizing methods that were self-directed such as encouraging, reassuring, validating, and empowering themselves. For example, Steve described dealing with his school-related stress by encouraging himself by saying to himself, “Okay, time to get this done because it’s due at midnight”. Additionally, Ross described validating his feelings of incompetence after an argument with his parents by telling himself that “Screw them, they don’t deserve you. You know what you’re worth and you know what you bring to the table and those relationships. Go make yourself feel better by doing something for yourself”.

### Urge to eat

Participants then elaborated on their experience of the urge to eat in the sub-themes below.

#### Reasons for wanting to eat

All participants in this study were guided to describe their urge to eat that they described as a tendency to want to escape their experiences or to replace their negative emotions with positive ones. For example, Ruth shared that “Ah, well, you know as you work your focused on your task. And I can be very focused has I work but when exhaustion sets in, I want to escape through food”. Raj shared that he ate to feel positive emotions by stating that “So I’m going to live my life and it doesn’t have to be completely disciplined. I have to enjoy it as well. It’s not a prison sentence. And that is whenever I try to eat food”.

#### Looking forward to the sensory experience

Five participants in this study described eating because they looked forward to the sensory experience of food. For example, Adam remarked that he looked forward to “… you know, the touch, the taste, the visual. It’s the whole thing for me”. Similarly, Stephanie described her excitement for food by sharing that “… you start to feel the smell of it and just the anticipation of it and again, how it’s going to taste when you eat it and like that feeling, you know the smell and the whole thing”.

### Eating

Participants in this study then described their experiences during eating, which are elaborated in the sub-themes below.

#### Overeating

Six participants in this study described feeling physically full but going back for more food. For example, Steve stated that “I had a meal to the point where I’d say like I was content. By no means, I was walking away from the meal being like ‘I’m not full.’ There’s definitely a sufficient amount”. Similarly, Ross stated that “I am grabbing the chips and bringing them back with a glass of water to the couch… And then it was after the first episode that I went back to the pantry again, at the beginning of the next episode. Pause after the beginning credits, go back”.

#### Positive feelings while eating

All participants in this study were guided to describe their feelings while eating that they described as positive experiences. For example, Fatima shared that “Well, ramen is comforting. Whenever I order ramen, it’s because it’s cold. I need something soothing. It’s like kind of like comfort food”. Similarly, Sofia shared that “I’m enjoying my drink while do my homework. And I feel relaxed”.

#### Enjoying the sensory experience

Five participants in this study described enjoying the sensory experience of food. For example, Stephanie shared, “… sight, texture, taste. It’s nice couple of… two small tortillas with jalapenos on there and cheese and it’s all crispy and smells great. And I’m like ‘Oh, wow. That looks good.’” Similarly, Sofia described enjoying the sensory experience of her effervescent tea in stating that “It makes me feel warm. I love sparkling water and I love something that’s effervescent”.

### How food or eating is helping me

In addition to describing what food or eating did for them (rewarding them and providing a coping mechanism; see Table 2), participants in this study also shared how food or eating helped them reward themselves or cope with difficult situations. These sub-themes are elaborated below.

#### Positively influencing my feelings

Six participants in this study shared that food or eating positively influenced their feelings. Some participants stated that eating enabled them to reduce their experiences of negative emotions. For example, Raj shared that “I would like to have two or three days, let’s say, in a month, where I can just eat and [get rid of] the stress”. Others stated that eating helped them to balance out their emotions. For example, Adam stated that “… it’s sort of balancing things out a middle, where the upset and the emotions… It’s just kind of leveling helping things level off a little”.

#### Not thinking about my stressors

Five participants described being able to not think about their stressors by eating. For example, Raj described the following while eating:

I’m not thinking about my life what will happen in the future or anything. Yeah. … That mental part, the cognitive part of what is going to happen in this… If I eat something, it will kind of engage that part. Maybe disguise that part.

Similarly, Steve shared that “And just like even sitting down eating food is kind of… I stopped pacing for a bit… or making food, definitely. And then eating it. I’ll stop pacing, because, again, I don’t just get intruded by thoughts”.

#### Being in the moment

Four participants also described being in the present moment while eating. For example, Steve stated that “I get like in the present moment [when] eating. It’s all very… I want to say passive but it’s not conscious. I just think about the texture, the flavour, what I’m eating…” Similarly, Raj shared that “It feels really good. I just want to close my eyes and enjoy that taste. Live in that moment”.

## Discussion

The current study explored the first-person subjective experience, or phenomenology, of emotional eating with a specific focus on the phenomenology of emotions and the role of food during emotional eating. Such an understanding is particularly important given that overeating is associated with an increased risk for developing overweight and obesity [18], as seen in six out of the eight participants in the current study who were overweight or obese. In addition, emotional eating has been linked to poorer psychological wellbeing, greater eating disorder symptoms, and higher levels of other psychopathology, such as depression [6, 12]). A total of 16 h of explicitation interviews were conducted with eight participants to allow them to access and verbalize the phenomenology of their emotional eating experiences. A thematic analysis of the data revealed nine themes describing the diachronic unfolding of the emotional eating experience and several subthemes describing the synchronic details of these moments. The core finding of this study is the phenomenological data supporting escape theory, namely that individuals tend to eat to replace their experience of negative emotions with pleasant feelings and sensory experience of food and to reduce associated ruminations and feel embodied in the present moment. Furthermore, during the experience of negative emotions, individuals try different behavioral, mental, and interpersonal methods to self-regulate but nevertheless turn to food, which may have been because they were unaware of their behavioral, interpersonal, or self-related psychological needs in moments of emotional eating or because they did not how to respond to them.

The current study is one of the few to explore the phenomenology of emotional eating. Previously, two other studies have explored the phenomenology of emotional eating. Kemp et al. [34] investigated the antecedent states and factors that lead to overeating by using open-ended, unstructured interviews and found that individuals ate to achieve short-term gratification from and to minimize their negative emotions. Similarly, Hernandez-Hons and Woolley [29] used semi-structured interviews to explore the phenomenology of attachment relationships and contextual factors that influence emotional eating to find that individuals used food to cope with insecure attachment patterns and for empowerment and acceptance. The current study advances the phenomenological exploration of emotional eating by using a more focused interview method, namely explicitation interviewing. Even though generalizability of study findings is not the expected attribute of qualitative research because of the focus on the subjective experience, the current study provides a few novel findings that future research can use meta-syntheses of qualitative research and quantitative methods to empirically investigate.

The core contribution of the current study is that it provides phenomenological evidence to support the escape theory of emotional eating introduced above and sets the stage to further the investigations of self-related processing proposed by this theory. According to escape theory, individuals who find it aversive to be aware of themselves or their current emotional states seek to disengage from higher-level meaning making involving thoughts or narratives about their identity or implications of certain events. Instead, they direct their awareness to lower levels of cognition wherein the self is reduced to the body, experience is reduced to sensation, and action is reduced to muscle movement. In the current study, the theme of “thinking a lot” in response to negative emotions that participants experienced is in line with this higher-order meaning-making. However, escape theory does not provide the necessary terminology to further explicate and empirically study these self-related processes. This has been provided elsewhere; for example, the higher-order processes described in escape theory and seen in the theme “thinking a lot” in this study have been referred to as a *narrative focus* involving linking subjective experiences through time using thoughts of self-traits, traits of others, memories of the past, and aspirations of the future [16]. On a broader level, this narrative focus can be mapped onto what Gallagher and Shear [19] describe as the narrative self, characterized by mental activities such as thoughts, emotions, and motivations, linked to one’s past and future. While such a mode is adaptive for cognitive and social functioning (such as through a central locus of control, sense of identity, etc., [53]), a narrative focus may be problematic if it is high in rumination, defined as a repetitive focus on symptoms of distress and their possible causes and consequences [25]. Particularly, high rumination activity has been shown to be associated with high negative affect and eating disorder symptomatology [35, 51]. In response to this narrative mode of functioning, individuals in the current study then described themes of “looking forward to the sensory experience” of food and consequently “enjoying the sensory experience”, “not thinking about my stressors”, and “being in the moment” while eating. This shift to sensory experience is in line with a lower level of cognition posited by escape theory and can be better understood as an *experiential focus* that involves inhibition of cognitive elaboration in favor of attending to present-moment sensory objects, including feelings and sensations rooted in the body [16]. Furthermore, this experiential focus can be mapped onto what Gallagher and Shear [19] describe as the minimal self, also referred to as the embodied self, because it consists of basic or core processes that are phenomenologically related to the body, such as sensorimotor experiences, perception of bodily boundaries, locus of self-reference, etc. Through the study of such variables related to the minimal self-related variables, it has been shown that this mode of functioning, when achieved without the use of food, is linked to positive wellbeing outcomes [13, 16, 24].

As such, the findings of the current study provide support for the proposition of escape theory that individuals emotionally eat to the shift their attention from higher to lower order cognition or, using specific self-related terminology, from the narrative self- to minimal self-related processing. Future empirical research is needed to validate the occurrence of these shifts in emotional eaters and their relationship to eating behaviors and emotion regulation in these individuals. Such an investigation would set the stage for the study of treatments that enable individuals to shift from narrative to minimal self-related processing without using food. One such practice that has been shown to enable a shift from narrative to minimal self-related processing without the use of food and the associated negative consequences of overeating is mindfulness, described as “paying attention in a particular way: on purpose, in the present moment, and non-judgmentally” [16, 32], p. 4. The efficacy of mindfulness practice in reducing emotional eating have already been established [33, 43]. Given that individuals with emotional eating attempt to shift self-related processing using food, future research could investigate whether such a shift in self-related processing without the use of food and instead as a mechanism of action of mindfulness occurs in individuals with emotional eating. Such an investigation would help us better understand the mechanisms underlying emotional eating that could be targeted by mindfulness-based programs to further improve their treatment efficacy.

The findings of the current study also showed a diversity in the needs of individuals in moments of emotional eating and the reasons why individuals were unable to meet their needs in these moments and instead turned to food. Specifically, the findings showed described different behavioral, interpersonal, and self-related needs without a large overlap among participants, thus highlighting the need for more individualized treatment of emotional eating. These needs of individuals seen in the current study are in line with previous research that showed the role of basic needs (as identified by Maslow’s hierarchy, such as physiological, love and belonging, self-esteem, etc.) in emotional eating and that the satisfaction of these needs was negatively correlated with intensity of emotional eating [54]. The negative correlation between needs satisfaction and emotional eating was replicated by another study that also showed that negative coping strategies mediated this relationship, thus highlighting the need for positive coping strategies for individuals to meet their needs and reduce emotional eating [2]. Furthermore, in our study, we found that the ability of individuals to meet their needs or cope positively with their negative emotions was hindered by a lack of awareness of their needs in the moments of emotional eating, impulsive emotional reactivity towards their needs, or negative ways that they related to themselves in moments of need, seen in subtheme “feelings, beliefs, and self-related reasons for not helping myself”. Evidence for the positive association of low interoceptive and emotional awareness, high impulsivity, and low self-esteem with high emotional eating has been shown in the literature [4, 9, 54, 56]. The findings from our study add to this work by specifying the mechanism by which low self-awareness, high impulsivity, and low self-esteem are linked with emotional eating, namely that these processes are some of the reasons individuals are unable to meet their needs during moments of negative emotions and thus turn to food instead. Future quantitative research using meditation analysis is needed to validate these findings. In addition to these intrapersonal variables, further research is needed to add to our understanding of how systemic issues such as racism (e.g., [30, 39]) influence individuals’ ability to cope with negative emotions.

The current study also provides phenomenological descriptions of ways in which individuals themselves attempted to cope with negative emotions without the use of food. This study is the first to provide such data because most research thus far has focused on teaching new skills to individuals with emotional eating and testing the efficacy of these interventions [18, 33]. Specifically, most individuals in this study described behavioral ways, such as moving away from the stimulus or taking a break, and cognitive ways, such as attention regulation strategies or distracting oneself, to regulate their negative emotions. In addition, the findings also showed the importance of social support through close others or a therapist and broadening one’s perspective, accepting external situations and oneself, and self-validating in reducing negative emotions. However, it is possible that individuals learned these methods from previous exposure to psychological interventions. In addition, it is unclear why these methods did not work for individuals in the current study because they eventually described emotionally eating after trying these methods. Even so, these findings of the methods used by individuals to cope with their difficult emotions without using food encourage the investigation of the efficacy of these methods in reducing emotional eating. Therapeutically, the behavioral and mental ways are central to treatment methods, such as Cognitive Behavioral Therapy (CBT, [17], and the self-related ways are central to programs that aim to increase acceptance and self-compassion, such as Acceptance and Commitment Therapy ACT, [26]. Research support for the use of these methods in helping individuals reducing their emotional eating is present [20, 41]. In addition, the therapeutic contact provided by all these treatment programs has been known to be a key factor in treatment, even in the treatment of eating disorders [22]. Future research could investigate some of the barriers that keep individuals from using these methods in moments of distressing emotional experiences and ways of making these skills more accessible to them during these moments.

The current study has a few limitations that must be considered in the interpretation of the findings. Firstly, even though care was taken to equally represent male and female-identifying participants, it may be useful to broaden the investigation to include a greater demographic variety and sexual orientation with a larger sample size. Secondly, the findings related to the self were guided by the interviewers’ questioning. As such, the central role played by the self in emotional eating needs further investigation because it is unclear whether participants would have elaborated on the role of the self, in addition to negative emotions, in exploring their emotional eating. Finally, all interviews in this study were conducted virtually over Zoom. As such, it is possible that some non-verbal and interpersonal factors that could facilitate a deeper exploration in the explicitation interviews were missing in this study. Future research could benefit from in-person explicitation interviews to further explore the phenomenology of emotional eating.

## Conclusion

The current study used explicitation interviewing to provide phenomenological support for the escape theory of emotional eating, which posits that individuals overeat to escape from higher levels of meaning making and associated aversive self-awareness to instead focus on lower levels of meaning and immediate bodily sensations. To advance the investigation of escape theory, specific self-related terminology is proposed, namely that individuals attempt to shift from narrative to embodied self-focus using food to regulate their negative emotions. Future research is encouraged to investigate these self-related processes in individuals with emotional eating to add to such support that exists for other populations. Such an investigation will support the recommendation of treatment programs, such as mindfulness-based interventions, that enable a shift in self-related processing without the use of food.

## Data Availability

Raw pseudonymized data is available on the Open Science Framework (https://doi.org/10.17605/OSF.IO/4AE5Z).
